# Supplementary material for: Predicting visual function by interpreting a neuronal wiring diagram
Source: Nature. 2024 Oct 2;634(8032):113–23. doi: 10.1038/s41586-024-07953-5 (PMC11446822; doi:10.1038/s41586-024-07953-5)
Supplement: Supplementary file 6 — CRF and ERF predictions for individual TmY4 and TmY9 cells. Analogous to Supplementary Data 3, but for TmY target types. Shown are the top four monosynaptic pathways, the strongest pathway passing through each of the top ten intermediary types (ranking from Extended Data Fig. 7), and the trisynaptic pathway Tm1–TmY–Dm3–TmY (see the section entitled Prediction of spatial normalization). [file 41586_2024_7953_MOESM6_ESM.zip › DataS4/TmY9q/720575940631488075.pdf]

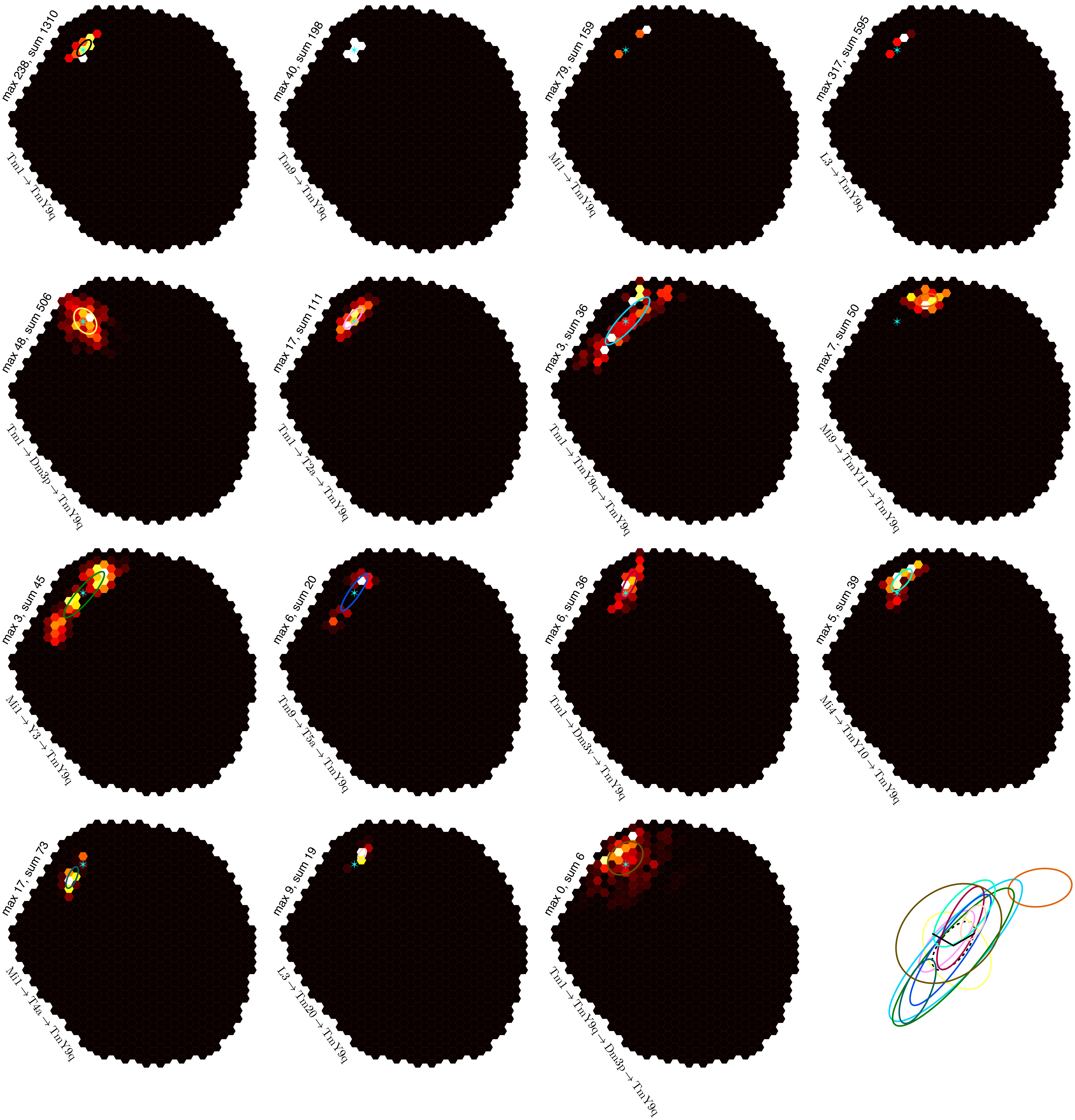
max 238, sum 1310
Tm1 → TmY9q
max 40, sum 198
Tm9 → TmY9q
max 79, sum 159
Mi1 → TmY9q
max 317, sum 595
L3 → TmY9q
max 48, sum 506
Tm1 → Dm3p → TmY9q
max 17, sum 111
Tm1 → T2a → TmY9q
max 3, sum 36
Tm1 → TmY9q → TmY9q
max 7, sum 50
Mi9 → TmY11 → TmY9q
max 3, sum 45
Mi1 → Y3 → TmY9q
max 6, sum 20
Tm9 → T5a → TmY9q
max 6, sum 36
Tm1 → Dm3v → TmY9q
max 5, sum 39
Mi4 → TmY10 → TmY9q
max 17, sum 73
Mi1 → T4a → TmY9q
max 9, sum 19
L3 → Tm20 → TmY9q
max 0, sum 6
Tm1 → TmY9q → Dm3p → TmY9q
